# The Potential of Phage Treatment to Inactivate Planktonic and Biofilm-Forming *Pseudomonas aeruginosa*

**DOI:** 10.3390/microorganisms12091795

**Published:** 2024-08-29

**Authors:** Inês Martinho, Márcia Braz, João Duarte, Ana Brás, Vanessa Oliveira, Newton C. M. Gomes, Carla Pereira, Adelaide Almeida

**Affiliations:** Department of Biology and CESAM, University of Aveiro, Campus Universitário de Santiago, 3810-193 Aveiro, Portugal; imartinho@ua.pt (I.M.); marciabraz96@ua.pt (M.B.); j.macedoduarte@ua.pt (J.D.); ana.bras12@hotmail.com (A.B.); and gomesncm@ua.pt (N.C.M.G.)

**Keywords:** *Pseudomonas aeruginosa*, phage therapy, biofilm, antibiotic-resistant bacteria, urinary tract infections

## Abstract

*Pseudomonas aeruginosa* is a common cause of hospital-acquired infections and exhibits a strong resistance to antibiotics. An alternative treatment option for bacterial infections is the use of bacteriophages (or phages). In this study, two distinct phages, VB_PaD_phPA-G (phPA-G) and VB_PaN_phPA-Intesti (phPA-Intesti), were used as single suspensions or in a phage cocktail to inactivate the planktonic cells and biofilms of *P. aeruginosa*. Preliminary experiments in culture medium showed that phage phPA-Intesti (reductions of 4.5–4.9 log CFU/mL) outperformed phPA-G (reductions of 0.6–2.6 log CFU/mL) and the phage cocktail (reduction of 4.2 log CFU/mL). Phage phPA-Intesti caused a maximum reduction of 5.5 log CFU/cm^2^ in the *P. aeruginosa* biofilm in urine after 4 h of incubation. The combination of phage phPA-Intesti and ciprofloxacin did not improve the efficacy of bacterial inactivation nor reduce the development of resistant mutants. However, the development of resistant bacteria was lower in the combined treatment with the phage and the antibiotic compared to treatment with the antibiotic alone. This phage lacks known toxins, virulence, antibiotic resistance, and integrase genes. Overall, the results suggest that the use of phage phPA-Intesti could be a potential approach to control urinary tract infections (UTIs), namely those caused by biofilm-producing and multidrug-resistant strains of *P. aeruginosa*.

## 1. Introduction

The discovery of penicillin marked a significant milestone in the history of medicine, beginning the golden era of antibiotic use [1]. However, the overuse and misuse of these drugs have resulted in the emergence of antibiotic-resistant bacteria [2]. The problem of antibiotic resistance has become a global health crisis, affecting our ability to effectively treat bacterial infections, and leading to increased morbidity and mortality and, consequently, increased economic costs associated with the treatments. In healthcare settings, antibiotic-resistant bacteria pose a greater risk due to invasive medical procedures and compromised immune systems [3]. *Pseudomonas aeruginosa* is one of the most common and serious causes of nosocomial infections, particularly affecting immunocompromised patients (especially neutropenic patients) and patients in intensive care units [4]. The Centers for Disease Control and Prevention (CDC) estimates that approximately 51,000 healthcare-associated infections caused by *P. aeruginosa* occur in the United States per year. Of these, 13% are multidrug-resistant (MDR), resulting in approximately 400 deaths per year [5]. In intensive care units across Europe, *P. aeruginosa* is the most frequent isolate in cases of pneumonia and is amongst the most frequently reported bacteria in urinary tract infections [6]. This bacterium is an opportunistic pathogen that can cause a variety of infections, including those of the skin and urinary tract, as well as more serious systemic infections such as pneumonia and sepsis [7,8]. The high mortality rates of *P. aeruginosa* infections reflect its acquired, intrinsic, or adaptive resistance to a range of antibiotics, including fluoroquinolones, beta-lactams, carbapenems, and aminoglycosides [9]. The mechanisms of *P. aeruginosa* resistance to antibiotics are diverse and include multidrug efflux systems, the production of β-lactamases, and low antibiotic permeability of the outer membrane [10,11,12]. *Pseudomonas aeruginosa* can also develop antibiotic resistance through mutation-driven mechanisms or horizontal gene transfer [13]. *Pseudomonas aeruginosa* uses the following two survival mechanisms: planktonic bacteria, which cause acute inflammation, and biofilm formation, which leads to persistent infections [14]. Autogenous extracellular polymeric substances constitute the primary composition of biofilms. This polymeric matrix envelops bacteria on surfaces and provides protection from environmental stresses [15,16]. Microbial biofilms adhere irreversibly to abiotic or biotic surfaces, protecting microorganisms from extreme conditions (such as host immune mechanisms in response to infection, environmental stress, and antimicrobial administration) [17]. The host immune response is initiated by the detection of various virulence factors present in microorganisms, which are crucial characteristics for the establishment of an infectious process and interaction with the host cells [18]. The investigation of biofilm-forming strains is of significant importance due to their heightened resistance to antimicrobial agents, the severity of infections they can cause in humans and animals due to their difficulty in eradication, and their capacity to survive on abiotic surfaces, including medical devices [19]. The antibiotic resistance of biofilms is significantly higher (ranging from 10 to 1000 times) than that of planktonic bacteria [20,21]. Therefore, new approaches are urgently needed to treat infections caused by MDR *P. aeruginosa*. One possible and promising alternative is phage therapy, which is emerging as a safe and effective treatment for bacterial infections [22,23,24,25,26].

Bacteriophages (or simply, phages) are viruses that only infect bacteria, taking over their metabolic machinery to replicate [27]. Phages can be broadly classified into the following two main types, according to their life cycle: virulent phages (recommended for phage therapy) and temperate phages (not recommended for phage therapy). Virulent phages, contrary to that of the temperate phages, have a strictly lytic life cycle, so they immediately replicate within their host cells, leading to cell lysis as well as the release of new phage particles which infect and cause the rapid death of other bacterial cells [28,29]. Phages have distinct advantages, when compared to antibiotics, including high specificity to target bacteria, low inherent toxicity, easy discovery in most environments, easy isolation, versatility in formulation and application, and also proven efficiency against biofilms [30,31,32,33]. Several studies have shown that phages can reduce biofilms due to their ability to penetrate them by lysing the different bacterial layers present in such biofilms [34,35,36,37,38]. Some phages encode in their genome polysaccharide depolymerases, which are specialized hydrolytic enzymes that target components of the bacterial cell wall, namely bacterial polysaccharides or polysaccharide derivatives [39]. These enzymes can degrade capsular polysaccharides of the biofilm matrix, enhancing phage adsorption and access to host cells [40]. On the other hand, the use of phages for biofilm eradication has some limitations, such as limited host range, high biofilm density, development of resistance to phages in biofilms, and inhibition of phage infection by quorum sensing in biofilms [41,42,43,44,45].

Phages have been investigated worldwide as a potential alternative to control *P. aeruginosa* infections [40,46,47,48,49,50,51,52], including MDR and biofilm formation strains involved in UTIs [53,54,55]. Ujmajuridze et al. (2018) showed that phage therapy could be effective and safe for the treatment of UTIs. In this study, six of the nine (67%) patients treated with an adapted Pyo phage revealed a decrease in the bacterial concentration (1.0–5.0 log). No phage-related adverse events were observed [55]. In another study, Khawaldeh et al. (2011) reported the efficacy of adjunctive phage therapy for a refractory *P. aeruginosa* UTI associated with bilateral ureteral stents and bladder ulceration, after repeated failure of treatments using only antibiotics. The patient was treated with pyophage #051007 instilled directly into the bladder daily, along with antibiotics (meropenem and colistin), which were administered from 6 days after the start of treatment. The patient was discharged from the hospital after 10 days, and the urine remained sterile for six months following the completion of the antibacterial treatment. During this period, the patient remained asymptomatic [53]. Other studies have also demonstrated that the combination of antibiotics and phages is more effective than either treatment alone in eradicating biofilms [41,42,43,44]. However, to our knowledge, there is only one study [56] that has evaluated the efficacy of the combination of antibiotics and phages in human urine.

The aim of this work was to evaluate the efficacy and safety of two phages to control *P. aeruginosa* in planktonic and biofilm cells. In addition, the potential impact of combining phage phPA-Intesti with the antibiotic, ciprofloxacin, on the inactivation of *P. aeruginosa* biofilms in human urine was evaluated to assess the phage’s potential for use in urinary tract infections and gain insight into its efficacy in the acidic environment of the urinary tract.

## 2. Materials and Methods

To study the potential application of phage therapy, two phages were isolated and their efficacy against an antibiotic-resistant strain of *P. aeruginosa* (CHUC) was evaluated. The kinetic theory of phage therapy postulates that the MOI is a crucial factor for an effective bacterial reduction [57,58,59]. Thus, bacterial reduction was initially determined in Tryptic Soy Broth (TSB; Liofilchem, Roseto degli Abruzzi, Italy) using phage phPA-Intesti and phPA-G at MOIs of 1, 10, and 100. The second in vitro assay, also conducted in TSB, was carried out with two phages combined in a cocktail (phPA-Intesti/phPA-G) at an MOI of 100. Then, the efficacy of phage phPA-Intesti at MOI 1 with or without antibiotic (ciprofloxacin at MIC) was evaluated on *P. aeruginosa* biofilms in urine. The full genome sequence of the phages was also analyzed to identify the presence of genes encoding the lysogenic markers, toxins, virulence, and antibiotic resistance genes.

### 2.1. Bacterial Strain and Growth Conditions

The antibiotic-resistant strain of *P. aeruginosa* (*P. aeruginosa* CHUC)*,* used in this study as a phage host, was a clinical isolate from a patient at the Centro Hospitalar e Universitário de Coimbra (CHUC). The other *P. aeruginosa* strains (PI24561, U96131, IR82433, IU4506, 2515567, IR83610, IU96174, IR80028, C563488, IR80722, IR87252, and IR77021) were isolated from Pedro Hispano Hospital patients, and ATCC 27853 was purchased from the American Type Culture Collection. These bacterial strains were grown on a solid medium, Tryptic Soy Agar (TSA, Liofilchem, Roseto degli Abruzzi, Italy), at 37 °C for 24 h and subsequently kept at 4 °C. Before each assay, one isolated colony was inoculated in 30 mL of Tryptic Soy Broth (Liofilchem, Roseto degli Abruzzi, Italy) and grown aerobically at 37 °C overnight, for 18–24 h, with stirring (120 rpm).

### 2.2. Phage Isolation and Preparation of Phage Stocks

Two distinct phages (phage phPA-G and phPA-Intesti) were used in this study.

Phage phPA-G was isolated from the sewage network of Aveiro (station EEIS9 of SIMRIA Multi Sanitation System of Ria de Aveiro), using the *P. aeruginosa* CHUC as host. Approximately 50 mL of sewage water was filtered through 0.45 µm pore size polycarbonate membranes (Millipore, Bedford, MA, USA) and added to 50 mL of a twice-concentrated TSB medium. A total of 500 µL of *P. aeruginosa* CHUC in the exponential growth phase was inoculated into the mixture, which was then incubated for 24 h at 37 °C and 50 rpm. Following the incubation period, the mixture was subjected to centrifugation at 10,000× *g* for 10 min at 4 °C. Filtration was then performed using a polyethersulfone layer filter with a 0.22 µm pore size (Merck-Millipore, Darmstadt, Germany). The filtrate was titrated according to the methodology outlined in [60] and stored at 4 °C. In brief, the filtrate preparation was subjected to a series of successive dilutions in phosphate-buffered saline (PBS) [137 mmol^−1^ NaCl (Sigma, St. Louis, MO, USA), 8.1 mmol^−1^ Na_2_HPO_4_.2H_2_O (Sigma, St. Louis MO, USA), 2.7 mmol^−1^ KCl (Chem-Lab NV, Zedelgem, Belgium), and 1.76 mmol^−1^ KH_2_PO_4_ (Applichem Panreac, Darmstadt, Germany), pH 7.4]. Next, 500 µL of each dilution, along with 200 µL of fresh bacterial culture, was added to 5 mL of molten TSA [30 g/L TSB (Liofilchem, Roseto degli Abruzzi, Italy), 6 g/L agar (Liofilchem, Roseto degli Abruzzi, Italy), 0.12 g/L MgSO_4_ (Sigma, St. Louis, MO, USA), and 0.05 g/L CaCl_2_ (Sigma, St. Louis, MO, USA), pH 7.4] and placed over a Petri plate containing solid TSA. Following a 24 h incubation period at 37 °C, a single phage plaque was isolated and transferred to PBS, followed by centrifugation. Subsequently, the resulting supernatant was employed as a phage source for a second isolation procedure. Four consecutive single-plaque isolation cycles were conducted to obtain a pure phage stock. All lysates were subjected to centrifugation at 10,000× *g* for 10 min at 4 °C in order to remove any bacteria or bacterial debris present. The phage phPA-G suspensions were kept at 4 °C.

Phage phPA-Intesti was isolated from a commercially available preparation, Intesti bacteriophage (Eliava BioPreparations Ltd., Tbilisi, Georgia), purchased from a pharmacy in Tbilisi, Georgia, using *P. aeruginosa* CHUC as a host. Successive dilutions of the “Intesti bacteriophage” were performed in PBS, and 500 µL of each dilution was added to 200 µL of fresh *P. aeruginosa* CHUC culture, mixed with 5 mL of molten TSA and then poured over a TSA plate. The plates were incubated at 37 °C for 24 h. One single phage plaque was selected from the solid medium and added to the TSB medium with a fresh *P. aeruginosa* CHUC culture. The mixture was then incubated for 16 h at 37 °C. Following centrifugation at 10,000× *g* for 10 min at 4 °C, the supernatant was employed as a source of phages for a subsequent isolation step. This process was repeated on three consecutive occasions, with each cycle involving single plaque isolation in order to obtain a pure phage stock. The resulting supernatant was subsequently filtered through a 0.22 μm pore size polyethersulfone layer (Merck-Millipore, Darmstadt, Germany). The phage phPA-Intesti suspensions were stored at 4 °C.

Phages phPA-G and phPA-Intesti stocks were propagated using the phage suspensions previously prepared in the SM buffer [0.1 M NaCl (Honeywell Fluka™, Seelze, Germany), 20 mM Tris-HCl (Sigma, St. Louis, MO, USA), and 8 mM MgSO_4_ (Sigma, St. Louis, MO, USA), pH 7.5]. The *P. aeruginosa* culture in the exponential growth phase underwent centrifugation at 10,000× *g* for 10 min, following which the pellet was resuspended in 50 mL of SM buffer. Then, 1 mL of the phage stock was added to 50 mL of SM buffer with bacteria. After incubation of the phage stocks for 24 h at 37 °C, with agitation at 50 rpm, the lysate was centrifuged at 10,000× *g* for 10 min at 4 °C. Then, the supernatant was filtered through a 0.22 µm pore size polyethersulfone layer (Merck-Millipore, Darmstadt, Germany). The phage suspension was kept at 4 °C, and the phage titer was determined through the double-layer agar method [60]. Plates were incubated at 37 °C for 24 h, and the lysis plaques were counted. The results of the phage titer were expressed as plaque-forming units (PFU)/mL. Phage suspensions were kept at 4 °C.

### 2.3. Electron Microscope Examination

A suspension of phages phPA-G and phPA-Intesti particles (10^9^ PFU/mL) was negatively stained with 2% uranyl acetate (Electron Microscopy Sciences, Hatfield, UK) for 1 min, and electron micrographs were taken using a JEOL 1011 Transmission Electron Microscope (TEM, JEDLUSA Inc, Peabody, MA, USA) operating at 100 kV. Images were acquired using a Gatan CCD-Erlangshen ES100W.

### 2.4. Phage Host Range and Efficiency of Plating

The host range of the phages phPA-G and phPA-Intesti was assessed by spot testing according to Duarte et al. (2021) [58] for the 14 bacterial strains listed in Table 1. Briefly, 300 µL of each bacterial strain was inoculated into 5 mL of molten TSA, which was then poured onto solid TSA plates and left to dried. Subsequently, 20 µL of each phage stock (10^9^ PFU/mL) was spotted over the bacterial lawn and the plates were incubated at 37 °C for 24 h before observation. After incubation, the susceptibility of each bacterial strain to the phages phPA-G and phPA-Intesti was assessed based on the presence or absence of a lysis zone. Bacterial sensitivity to the phages was inferred from the presence of lysis zones (+) or the absence of clear lysis zones (−).

The EOP was determined for the bacterial strains with the presence of a lysis zone, using the double-layer method [60]. Briefly, successive dilutions of the phage stock (10^9^ PFU/mL) were performed in PBS. A total of 500 µL of each dilution (10^0^–10^−7^), along with 200 µL of fresh of bacteria strain, was added to 5 mL of molten TSA and placed over a Petri plate containing solid TSA. Plates were incubated at 37 °C and observed for the presence of lytic plaques after 24 h. Phage plaques were counted, and the results were expressed in PFU/mL. The EOP was calculated as the ratio between the PFU/mL on the target bacterial strain and the PFU/mL on the host strain (*P. aeruginosa* CHUC). An EOP value of 1 indicates that the phage is equally efficient on both target and host strains. An EOP of less than 1 indicates that the phage is less efficient on the target strain, compared to the host strain, and an EOP of greater than 1 indicates that the phage is more efficient on the target strain [60]. An EOP value of 0 indicates that the phage does not infect the tested bacterial strain (absence of clear lysis zones).

### 2.5. In Vitro Kill Curves with Planktonic Bacteria

The inactivation of *P. aeruginosa* CHUC in TSB (with a final concentration of 10^5^ CFU/mL) by phages phPA-G and phPA-Intesti was evaluated at MOIs of 1, 10, and 100 (final concentrations of 10^5^, 10^6^, and 10^7^ PFU/mL, respectively). The efficiency of the phage cocktail, phPA-G, and phPA-Intesti was evaluated at an MOI of 100. Two control groups were also included in each assay—a bacterial control inoculated with only *P. aeruginosa* CHUC (BC) and a phage control inoculated with only the phage (PC). For the phage cocktail experiments, the cups were inoculated with phages from their respective stocks whose titer had been determined prior to each assay. Aliquots of the test and control groups were collected at 0, 2, 4, 6, 8, and 12 h after inoculation. The bacterial concentration was determined in triplicate in solid TSA medium by the drop-plate technique [61] after a 24 h incubation at 37 °C. The phage titer was determined in triplicate by the double-layer agar method, as described by [60], following an incubation period of 24 h at 37 °C. Three independent assays were conducted for each condition.

### 2.6. Phage DNA Extraction

The nucleic acid of the virion was extracted using the ZymoBIOMICS™ DNA Miniprep Kit (Zymo Research, Irvine, CA, USA), following the manufacturer’s protocol. The DNA quantity was then measured with a Qubit 3.0 Fluorometer (Thermo Scientific, Waltham, MA, USA) using the Qubit 1X dsDNA assay.

### 2.7. Phage Genome, Assembly and Annotation

Phage genome sequencing was conducted by SeqCenter, LLC (Pittsburgh, PA 15201, USA). Illumina sequencing libraries were prepared using the tagmentation-based and PCR-based Illumina DNA Prep kit with custom IDT 10 bp unique dual indices (UDI) targeting an insert size of 320 bp. No additional DNA fragmentation or size selection steps were necessary. Sequencing was performed on an Illumina NovaSeq 6000, producing 2 × 151 bp paired-end reads in one or more multiplexed shared-flow-cell runs. Demultiplexing, quality control, and adapter trimming were performed using bcl-convert (v4.1.5). Quality control of the raw sequence reads was carried out with FASTQC v0.11.9 (Bioinformatics Group at the Babraham Institute, Cambridge UK), both before and after trimming the low-quality reads with Trimmomatic v.0.39 using the following parameters: LEADING: 8; TRAILING: 8; SLIDINGWINDOW: 4:15; and MINLEN: 100 [62]. The trimmed reads were assembled de novo using SPAdes v.3.13.1, with the -careful parameter [63]. Assembly graphs were inspected using Bandage v0.8.1 [64]. Reads were mapped back to the resulting assembly using BBMap v38.18 to determine the average coverage of each contig [65]. Manual filtering removed contigs with dubious coverage. If necessary, reads were remapped to the filtered contig file using Bowtie2 v.2.5.1 [66] and assembled with SPAdes v.3.13.1. Further correction and polishing of the assembly errors were conducted using Pilon v1.24 [67]. Termini prediction using PhageTerm [65] was unsuccessful; however, the genomes were determined to be circularly permuted using apc.pl (https://github.com/jfass/apc, download at 22 November 2023), and any repeated sequence artifacts were removed. The phage genomes were manually reordered to match the most closely related phage based on the method described by Shen and Millard (2021) [68]. The full genome sequences were compared to those in GenBank using BLASTN (somewhat similar sequences), identifying the most closely related phages. The completeness and contamination of the phage genomes were assessed using CheckV v1.0.1 [69]. Annotation of the phage genomes for the coding DNA sequences (CDS), tRNA, tmRNA, CRISPRs, virulence factors (VFs), toxins, and antimicrobial resistance genes (ARGs) was performed using Pharokka v1.3.2 [68]. The CDS were assigned to functional categories using PHROGs [70].

### 2.8. Urine Sample Collection and Handling

Urine samples were collected from the same individual following the protocol of the Laboratory of Clinical Analysis Avelab (Aveiro, Portugal) as described by [71]. Subsequently, after collection, the samples were transported to the laboratory, centrifuged at 10,000× *g* for 10 min and filtered using a 0.22 µm pore size filter into sterile containers to be used later in the assay. All urine samples were collected from the same individual at similar hours to reduce variability. The urine samples presented a pH of about 6 and were not further analyzed, being used only as a culture media. Thus, ethical issues are not applicable.

### 2.9. Biofilm Formation and Quantification in Urine

Prior to each assay, *Pseudomonas aeruginosa* CHUC was inoculated into a fresh TSB medium and incubated at 37 °C with orbital shaking (120 rpm) for 24 h. After incubation, 300 μL of the grown bacterial suspension was transferred to 30 mL of urine. The urine was collected immediately before the experiment and filtered through 0.22 µm pore size polyethersulfone membranes (Millipore, Bedford, MA, USA). Then, 200 μL of the urine inoculated with *P. aeruginosa* was transferred to each well of a 96-well plate. The plates were incubated at 37 °C for 24 h without agitation. After incubation, suspended cells were removed, and the wells were washed three times with PBS to remove all planktonic or non-adherent cells. Following this, the wells were replenished with fresh urine. For biofilm quantification, the plate (containing 200 μL of fresh urine) was sonicated (BANDELIN, SONOREX SUPER RK 102 H) for 10 min. Aliquots were then collected, serially diluted in PBS, and subsequently plated by the drop-plate method (two drops of 10 μL each) onto plates containing TSA [61]. The plates were incubated at 37 °C for 24 h. After incubation, bacterial colonies were counted, and the results were expressed in CFU/cm^2^.

### 2.10. Determination of the Minimum Inhibitory Concentration (MIC) of Ciprofloxacin

The concentration of *P. aeruginosa* cultures was adjusted to the turbidity of 0.5 McFarland standards in Mueller Hinton Broth (Liofilchem, Roseto degli Abruzzi, Italy) to give a bacterial concentration of 5 × 10^7^ CFU/mL. Bacterial cultures were adjusted to 5 × 10^5^ CFU/mL. A range of concentrations from 75 μg/mL to 0.14 μg/mL of ciprofloxacin (Cip, Sigma-Aldrich, St. Louis, MO, USA) was tested. An equal volume (100 µL) of the antibiotic and the standardized inoculum (5 × 10^5^ CFU/mL) was mixed in the 96-well plates. A control sample of bacteria without antibiotics was also included. The plates were incubated for 24 h at 37 °C without agitation. The MIC was established as the lowest concentration of ciprofloxacin with no visible bacterial growth. Three independent assays were conducted for each condition.

### 2.11. Biofilm Reduction in Urine Using Phage and/or Ciprofloxacin

The most effective phage (phPA-Intesti) on planktonic bacteria (see Section 2.5) was tested on the *P. aeruginosa* biofilms in urine, alone or in combination with ciprofloxacin. Biofilm formation was performed in 96-well plates as described in Section 2.8. Phage phPA-Intesti was added to the bacterial biofilm at a final concentration of 10^7^ PFU/mL to achieve an MOI of 1. Ciprofloxacin was added to bacterial biofilm at the MIC.

Five groups were included as follows: a bacterial control containing only the bacterial biofilm (BC), a phage control containing only the phage phPA-Intesti (PC), a control containing the phage and antibiotic (PC + Cip), an antibiotic sample containing the *P. aeruginosa* biofilm and antibiotic (Cip), a sample containing the *P. aeruginosa* biofilm and the phage (BP), and a sample containing the *P. aeruginosa* biofilm, antibiotic, and phage (BP + Cip). All groups were incubated for 24 h at 37 °C without agitation. Aliquots of the test samples and controls were collected at 0, 4, 8, and 12 h. The quantification of phage and bacteria was performed as described in Section 2.5. Three independent experiments were performed, with three replicates in each condition.

### 2.12. Statistical Analysis

The statistical analysis was conducted using GraphPad Prism 10. The Kolmogorov–Smirnov test was employed to assess the normality of distributions, while the Levene test was used to determine the homogeneity of variances. The significance of the differences between the control and treatment groups was evaluated using a two-way ANOVA test. A value of *p* < 0.05 was considered to be statistically significant.

## 3. Results

### 3.1. Electron Microscope Examination

**Figure 1:**
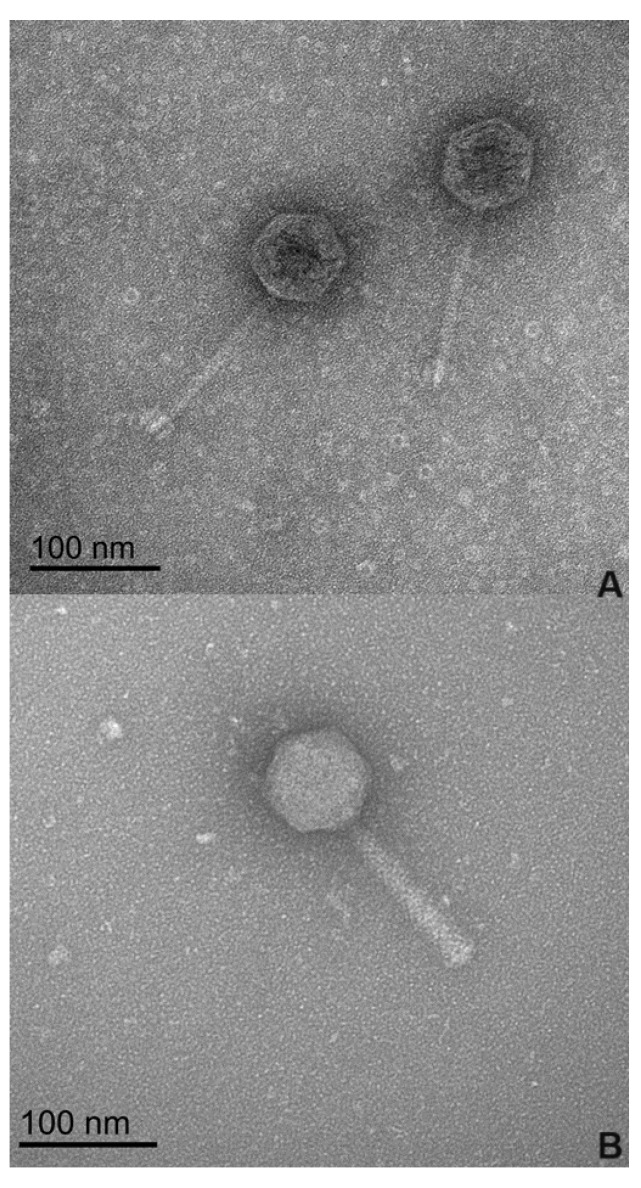
Electron microscopy images of the two phages at 100 nm magnification. (**A**) Phage phPA-G; (**B**) Phage phPA-Intesti.

The morphological analysis of the phages by transmission electron microscopy (TEM) (Figure 1), revealed that phages phPA-G and phPA-Intesti exhibit siphovirus and myovirus morphotypes, respectively, and are classified as *Caudoviricetes*. Phage phPA-G has an icosahedral head with an approximately 64 ± 2 nm width and a long tail with a length of approximately 123 ± 3 nm. Phage phPA-Intesti has an elongated icosahedral head with an approximately 68 ± 1 nm width and a contractile tail with a length of approximately 122 ± 2 nm.

### 3.2. Phage Host Range Determination and Efficiency of Plating (EOP) Analysis

The spot test showed that phage phPA-Intesti was able to form clear zones on 5 of the 14 strains tested (35.7% of the tested strains). However, EOP results indicated that phPA-Intesti formed phage lysis plaques beyond the host only in two strains (*P. aeruginosa* IR82433 and IR83610), with an EOP of 3.1 × 10^−2^ and 4.3 × 10^−1^, respectively. Phage phPA-G only infected its host (*P. aeruginosa* CHUC).

### 3.3. In Vitro Kill Curves with Planktonic Bacteria

**Figure 2:**
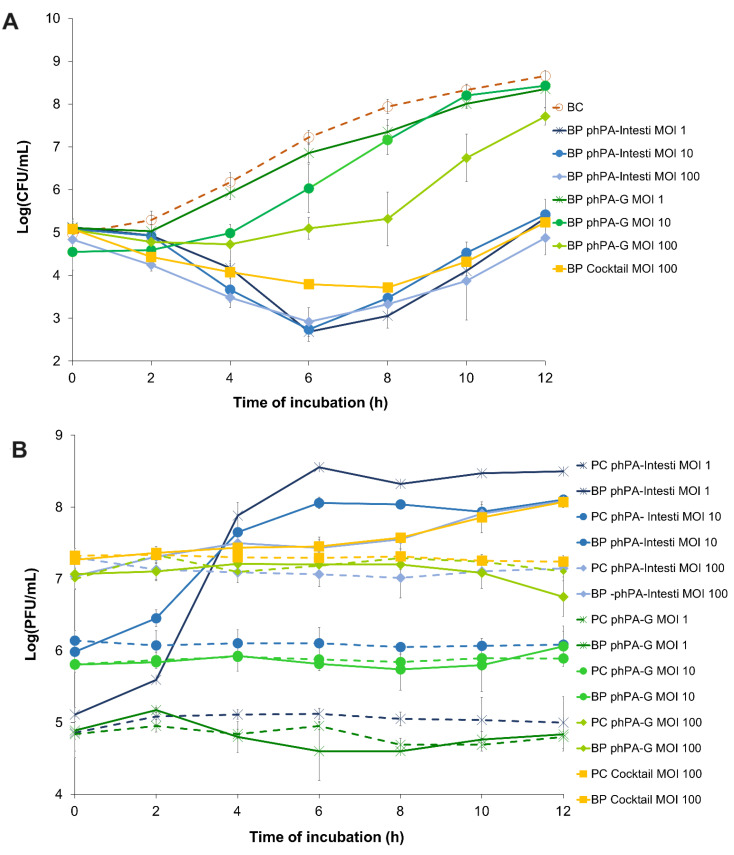
The effect of two phages alone (phPA-G and phPA-Intesti) at MOI 1, 10, and 100, and phage cocktail (phPA-G/phPA-Intesti) at MOI 100 against *P. aeruginosa* CHUC during 12 h of incubation. (**A**) Bacterial concentration: BC—bacteria control; BP—bacteria plus phage. (**B**) Phage concentration: PC—phage control; BP—bacteria plus phage. Values represent the mean of three independent assays, with three replicates in each condition. Error bars represent the standard deviation.

The bacterial density in the bacterial control (BC) increased by 4.2 log CFU/mL (ANOVA, *p* < 0.05, Figure 2A) during the 24 h incubation.

The maximum bacterial inactivation with phage phPA-G was 2.6 log CFU/mL (Figure 2A), which was achieved after 8 h of incubation (ANOVA, *p* < 0.05) for the multiplicity of infection (MOI) of 100, when compared with the bacterial control. However, after 4 and 6 h of incubation, the rate of inactivation was already considerably high for phage phPA-G (reduction of 1.5 and 2.1 log CFU/mL, respectively) when compared with the bacterial control (ANOVA, *p* < 0.05, Figure 2A). Despite bacterial regrowth, the differences in bacterial density between BC and the test group (BP phPA-G MOI 100) remained statistically significant (ANOVA, *p* < 0.05, Figure 2A) until the end of the experiment (decrease of 0.9 log CFU/mL after 12 h of incubation). No statistically significant differences were detected between MOI 1 and the bacterial control during treatment (ANOVA, *p* > 0.05, Figure 2A). At an MOI of 10, only after 4 and 6 h of incubation, significant differences in bacterial concentrations were observed (ANOVA, *p* < 0.05), compared to the bacterial control (reductions of 1.2 and 1.2 log CFU/mL, respectively). In general, the bacterial reduction achieved at an MOI of 100 with phage phPA-G was significantly higher than that of the other MOIs tested. However, after 2 h of incubation, no significant differences were observed between the MOIs tested. After 4 h of incubation, the bacterial reduction was similar for MOIs 10 and 100. However, statistically significant differences were observed when comparing MOIs 1 and 10 (decreases of 0.3 and 1.2 log CFU/mL, respectively) and MOIs 1 and 100 (decreases of 0.3 and 1.6 log CFU/mL, respectively) (Figure 2A).

The maximum bacterial reduction with phage phPA-Intesti was 4.9, 4.5, and 4.7 log CFU/mL, for MOIs 1, 10, and 100, respectively, observed after 8 h of incubation (ANOVA, *p* < 0.05, Figure 2A), when compared with bacterial control.

Following 4 h of incubation, the bacterial reduction was already significantly high for phage phPA-Intesti (reductions of 2.9, 2.5, and 2.8 log CFU/mL for MOIs 1, 10, and 100, respectively) when compared with bacterial control (ANOVA, *p* < 0.05, Figure 2A). After 6 h of incubation, *P. aeruginosa* regrowth occurred in the phage-treated groups at all MOIs. Despite this bacterial regrowth, the differences in bacterial density between the control group (BC) and test groups (BP phPA-Intesti MOI 1, MOI 10, and MOI 100) remained statistically significant (ANOVA, *p* < 0.05, Figure 2A) until the end of the experiment (reduction of 3.3, 3.2, and 3.9 log CFU/mL for MOIs 1, 10, and 100, respectively). No statistically significant differences were observed between MOI 1 and 10 after 2, 6, 8, and 12 h of incubation (ANOVA, *p* > 0.05). When comparing inactivation at MOIs of 1 and 100, only after 2 and 4 h of incubation differences in bacterial counts were observed. At MOI of 100, after 2, 10, and 12 h of incubation, the rate of reduction was significantly higher (ANOVA, *p* < 0.05, Figure 2A) than the one obtained with the MOI of 10.

Phages phPA-Intesti and phPA-G did not present any significant variations in titer during 12 h of incubation in the control groups (PC) for all the tested MOIs (ANOVA, *p* > 0.05, Figure 2B). No significant differences were observed for phage phPA-G when incubated with the host *P. aeruginosa* at an MOI of 1, 10, and 100 compared to the phage control (ANOVA, *p* > 0.05, Figure 2B). When phage phPA-Intesti was incubated in the presence of the host *P. aeruginosa* CHUC, a significant increase in phage titer was achieved after 12 h of incubation (increase of 3.5, 2.0, and 0.9 log PFU/mL for MOIs 1, 10, and 100, respectively) (ANOVA, *p* < 0.05), in comparison with the phage control (Figure 2B). The maximum of *P. aeruginosa* inactivation with phage cocktail (phPA-G/phPA-Intesti) was 4.2 log CFU/mL, achieved after 8 h of incubation (ANOVA, *p* < 0.05). However, after incubating for 4 and 6 h, the bacterial reduction was already significantly high for the phage cocktail (a decrease of 2.1 and 3.4 log CFU/mL, respectively) when compared with the bacterial control (ANOVA, *p* < 0.05, Figure 2A). Bacterial regrowth was observed after 8 h of incubation, but the differences in bacterial density between the control group (BC) and the phage cocktail-treated group (BP phage cocktail MOI 100) remained statistically significant (ANOVA, *p* < 0.05, Figure 2A) until the end of the experiment (reduction of 3.4 log CFU/mL).

When comparing the inactivation between the two phages, the rate of bacterial inactivation with phage phPA-Intesti was significantly higher than the one obtained with the phage phPA-G (ANOVA, *p* < 0.05) for all the MOIs studied. However, after 2 h of incubation, no statistically significant differences were detected between phages phPA-Intesti and phPA-G at an MOI of 1, 10, and 100 (ANOVA, *p* > 0.05) during treatment. In general, the bacterial inactivation of phage phPA-Intesti and the phage cocktail was similar (ANOVA, *p* > 0.05; Figure 2A) during treatment. However, after 6 h of incubation, the rate of bacterial inactivation with phage phPA-Intesti (reduction of 4.5 log CFU/mL) was significantly higher (ANOVA, *p* < 0.05) than that of the phage cocktail (reduction of 3.4 log CFU/mL). During the first 4 h of incubation, no statistically significant differences were detected between phage phPA-G and the phage cocktail at an MOI of 100. However, after this period of incubation, the rate of bacterial inactivation with the phage cocktail was significantly higher (ANOVA, *p* < 0.05) than that of phage phPA-G.

The phage cocktail remained constant throughout the experimental period in the control group (PC) (ANOVA, *p* > 0.05, Figure 2B). When the phage cocktail was incubated with the host (BP cocktail MOI 100), a significant increase of 0.8 log PFU/mL was observed after 12 h of incubation (ANOVA, *p* < 0.05), when compared with the phage control (PC cocktail MOI 100, Figure 2B).

### 3.4. Genome Analysis

**Figure 3:**
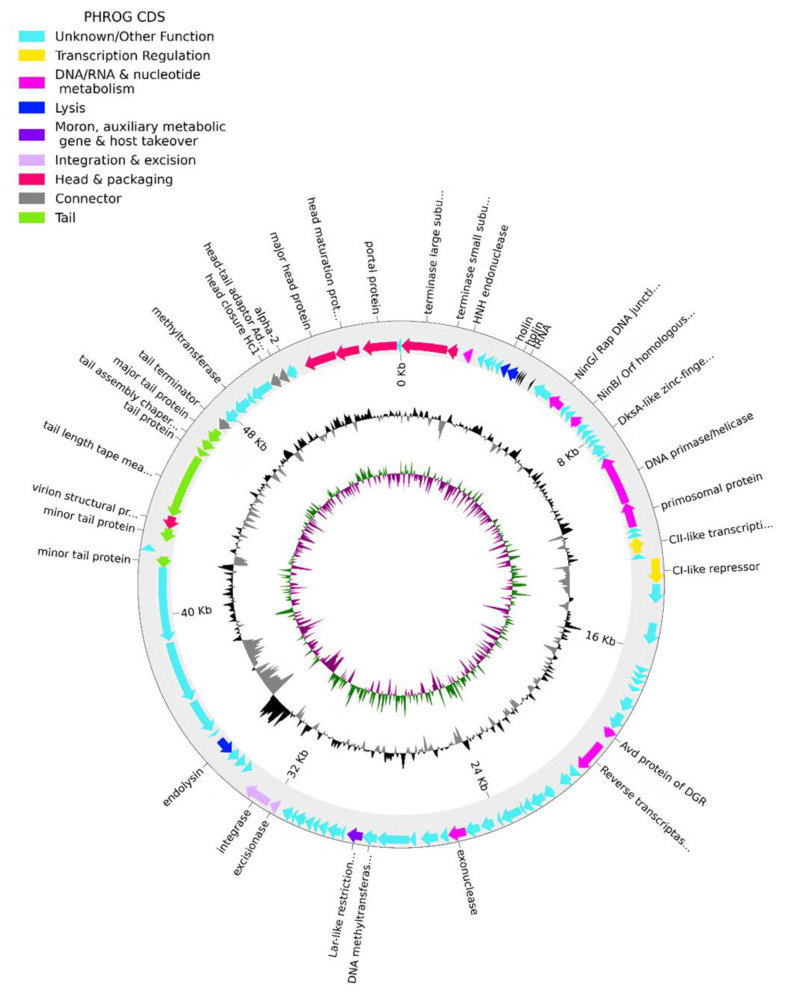
Phage phPA-G genome map. The outer circle with arrow-headed bands, depicts the coding DNA sequences (CDS), with colors indicating the functional categories of the predicted genes in the transcription direction. The innermost ring depicts the genome GC skew (green/purple), followed by GC content (black/grey). The labels indicate the predicted functions of the CDS, which are color-coded according to the PHROGs categories.

The genome of phPA-G (Genbank: PP083311) is a double-stranded DNA molecule of 54,834 bp, and the average GC content is 58.5%. The CheckV results showed 98.5% of completeness and no host contamination. The genome contained 92 predicted coding DNA sequences (CDSs) and 5 tRNAs. Among the 92 CDSs, 36 had been assigned to functional genes and the remaining 56 were annotated as hypothetical proteins (Table S1). The genome of phage phPA-G harbors a diverse array of genes, including those involved in DNA replication and modification, transcriptional regulation, phage packaging and structural proteins, and proteins associated with host lysis (Figure 3, Table S1).

More specifically, phage phPA-G possesses genes coding for lysogenic markers, such as an integrase and an excisionase, suggesting its potential for establishing a latent infection within host cells (Figure 3, Table S1). However, the phage phPA-G lacks genes coding known toxins, antimicrobial-resistance genes (ARGs), virulent factors (VFs) of bacterial origin, tmRNA, or CRISPRs (Table S1).

**Figure 4:**
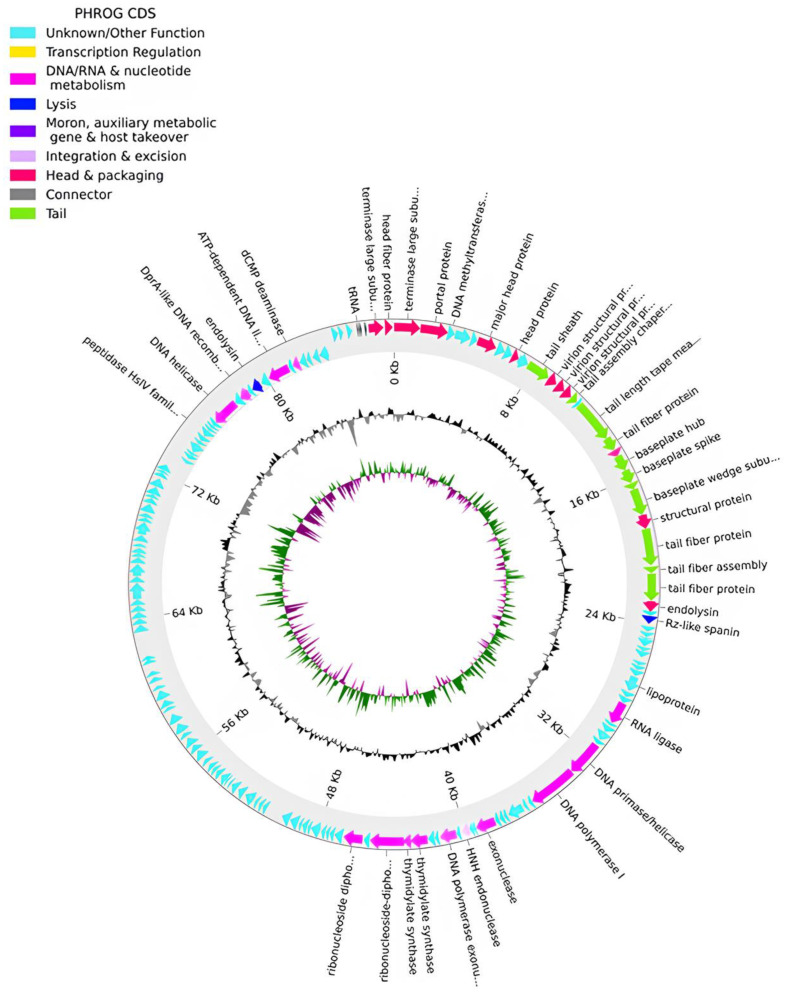
Phage phPA-Intesti genome map. The outer circle, comprising arrow-headed bands, depicts the coding DNA sequences (CDS), with colors indicating the functional categories of the predicted genes in the transcription direction. The innermost ring depicts the genome GC skew (green/purple) followed by GC content (black/grey). The labels indicate the predicted functions of the CDS, which are color-coded according to the PHROGs categories.

The genome of phage phPA-Intesti (Genbank: PP083312) is constituted by a double-stranded DNA of 87,746 bp in length and a GC content of 54.8%. The CheckV results showed 100% completeness and no host contamination. A total of 157 CDSs were annotated, of which approximately 73% were classified as having hypothetical functions. Our analysis identified putative genes coding for three tRNAs and essential components of the phage’s replication machinery, metabolism, structure, DNA packaging, and cell lysis systems (Figure 4, Table S2). No genes coding for lysogenic markers were detected in the genome of this phage. Similar to phPA-G, phage phPA-Intesti lacked known genes coding for VFs, ARGs, tmRNA, or CRISPRs (Table S2).

The genome-wide BLAST analysis revealed that phPA-G belongs to the genus *Detrevirus* and phPA-Intesti belongs to the genus *Nankokuvirus*. The genome sequence of phage phPA-G showed a 96.20% similarity to phage vB_Pae_BR153a and a 96.88% similarity to phage vB_PeaS_FBPa47, while the genome coverage was only 21% for phage vB_Pae_BR153a and 51% for phage vB_PeaS_FBPa47. The genome sequence of phage phPA-Intesti had strong similarity with the *Pseudomonas* phages vB_PaeM_RP6, vB_PaeM_RP7, and vB_PaeM_RP8 (Coverage: 100%, Identity: 100%, GenBank Accession No. ON524862, ON524863 and ON524864, respectively).

### 3.5. Determination of Minimum Inhibitory Concentration (MIC)

The assessed MIC of the *P. aeruginosa* CHUC to ciprofloxacin was 0.58 µg/mL. *Pseudomonas aeruginosa* CHUC was susceptible to ciprofloxacin, according to EUCAST criteria (resistance breakpoint, 0.5 mg/mL) [72].

### 3.6. Biofilm Reduction Assays in Urine Using Phage and/or Antibiotic

**Figure 5:**
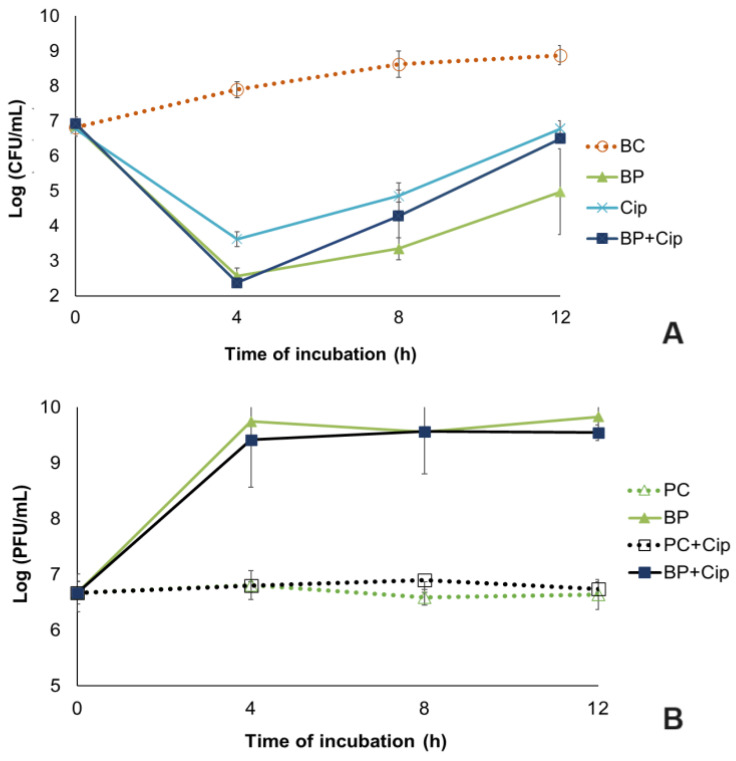
*Pseudomonas aeruginosa* biofilm reduction by the phPA-Intesti at MOI 1 and/or ciprofloxacin at MIC concentration in urine during a 12 h incubation. (**A**) Bacteria concentration: BC—biofilm control (only bacteria); BP—biofilm + phage; Cip—biofilm + ciprofloxacin; BP + Cip—biofilm + phage + ciprofloxacin; (**B**) Phage concentration: PC—phage control (only phage); PC + Cip—phage + ciprofloxacin; BP—biofilm + phage; BP + Cip—biofilm + phage + ciprofloxacin. Values represent the mean of three independent assays, with three replicates in each condition. Error bars represent the standard deviation.

In the bacteria control group (BC), the biofilm cell density increased by 2.1 log CFU/cm^2^ (ANOVA, *p* < 0.05, Figure 5A) during 12 h of the experiment.

When comparing the biofilm cell density in the phage phPA-Intesti-treated group with the bacterial control, a maximum reduction of 5.3 log CFU/cm^2^ was achieved after 4 h of experiment (ANOVA, *p* < 0.05, Figure 5A). Following the incubation period, biofilm cell regrowth was observed in the phage-treated group. However, the differences in bacterial concentration between the phage-treated group and the bacterial control remained statistically significant (ANOVA, *p* < 0.05, Figure 5A) until the end of the experiment (decrease of 5.3 and 3.9 log CFU/cm^2^ after 8 and 12 h of incubation, respectively).

The maximum bacterial inactivation when phage phPA-Intesti was combined with ciprofloxacin at the MIC (BP + Cip) relative to the bacterial control was 5.5 log CFU/cm^2^, achieved after 4 h of treatment (Figure 5A). In general, the inactivation of biofilm cells was significantly higher (ANOVA, *p* < 0.05) in the phage treatment (BP) than in the antibiotic treatment, with (BP + Cip) or without the phage (Cip). However, after 4 h of inactivation, no statistically significant differences (ANOVA, *p* > 0.05, Figure 5A) were observed between phage treatment (BP) and phage plus ciprofloxacin treatment (BP + Cip) (reduction of 5.3 and 5.5 log CFU/cm^2^, respectively).

The maximum reduction in biofilm cells in the antibiotic treatment was 4.3 log CFU/cm^2^, achieved after 4 h of treatment, which was significantly lower (ANOVA, *p* < 0.05) than that which was observed when the phage and antibiotic were combined (reduction of 5.5 log CFU/cm^2^). After this period, no statistically significant differences (ANOVA, *p* > 0.05, Figure 5A) were observed between antibiotic treatment (Cip) and phage plus ciprofloxacin treatment (BP + Cip). When phage treatment (BP) was used, bacterial regrowth was generally lower than when the other treatments (Cip and BP + Cip) were tested. Despite this bacterial regrowth, the differences in bacterial concentration between the phage-treated group (BP, Cip and BP + Cip) and bacterial control group (BC) were statistically significant (ANOVA, *p* < 0.05, Figure 5A) until the end of the assay.

The phage titer in the controls (PC and PC + Cip) remained constant throughout the assay (ANOVA, *p* > 0.05, Figure 5B). When the phage was incubated in the presence of the *P. aeruginosa* (BP) and the antibiotic (BP + Cip), a significant increase of 3.2 and 2.8 log PFU/cm^2^, respectively, was observed after 12 h of incubation (ANOVA, *p* < 0.05, Figure 5B), when compared with the phage control (PC) and phage + antibiotic control (PC + Cip), respectively.

## 4. Discussion

Antimicrobial research currently faces significant challenges in the discovery and development of new antibiotics. Despite numerous strategies aimed at developing new antimicrobial drugs, the emergence of bacterial resistance is inevitable. As a result, pharmaceutical companies that invest resources in antibiotic development may experience reduced profits, making them reluctant to invest further in the sector [73]. This has led to a significant gap between the development of antibiotics and the emergence of antibiotic-resistant bacteria [2]. Therefore, current research is aimed at finding innovative and alternative approaches to bacterial infectious diseases. Several studies have presented phages as an alternative treatment against *P. aeruginosa* [40,46,47,48,49,50,51,52,53,54,55,74,75]. However, despite the success of phage preparations, most of the isolated and characterized phages exhibit a very narrow host range, infecting only one bacterial genus or even particular strains [76]. The implementation of phage libraries can address this limitation, allowing for the selection of effective phage suspensions for a specific strain [77]. Since infections caused by multidrug-resistant and biofilm-forming strains of *P. aeruginosa* continue to increase worldwide [45], the selection and evaluation of the efficacy of new phages have become crucial for providing new and effective treatment options for patients, such as those with UTIs. According to Tagliaferri et al. (2019) [78], the combination of two or more agents with different mechanisms of action should enhance bacterial inhibition, broaden the spectrum of action, and overcome defense mechanisms, such as biofilm formation. Some studies have demonstrated that the combination of antibiotics and phages is more effective than either treatment alone in eradicating biofilms [41,42,43,44]. However, some studies have shown that ciprofloxacin can have synergetic [41,79], antagonistic [80,81], and neutral [82] behaviors when combined with phages. To our knowledge, there is only one study [56] that has evaluated the efficacy of the combination of antibiotics and phages in human urine. Thus, in this study, two phages (phPA-G and phPA-Intesti) alone or combined in a phage cocktail were used to control *P. aeruginosa* in planktonic and biofilm cells, and the potential effect of combining the phage phPA-Intesti with ciprofloxacin against *P. aeruginosa* biofilms in urine was also evaluated. The results showed the following: (1) phage phPA-Intesti was more efficient than phage phPA-G; (2) the combination of phages in a cocktail did not improve the efficacy of bacterial inactivation; (3) phage treatment was more effective than antibiotic treatment; (4) the combination of phage phPA-Intesti with ciprofloxacin did not increase *P. aeruginosa* inactivation; and (5) the combination of phage and antibiotic did not reduce the development of bacterial resistance to the phage.

The specificity of phages is of great importance in the context of phage therapy, as evidenced by the results of the two phages tested in this study. Phage phPA-G exhibited high specificity, with a narrow host spectrum, infecting only the original host bacterium (*P. aeruginosa* CHUC). The narrow host range of phage phPA-G may be due to the presence of genes coding for lysogenic markers that influence phage replication against the host (the number of phage particles in the presence of the host remained constant during 12 h of incubation) and other *P. aeruginosa* strains. However, phage phPA-Intesti, apart from their host (*P. aeruginosa* CHUC), can also infect 2 other *P. aeruginosa* strains of the 13 strains tested in this study. The absence of lysis observed in several *P. aeruginosa* strains may also be explained by variations in the phage’s recognition process, which can be different from strain to strain due to the different expression of phage receptors on the bacterial outer membrane surface. Phages differ substantially in the bacterial hosts that they infect. Their host range is determined by the specific structures that they use to target bacterial cells, namely phage tail fibers (or spikes), which initially mediate reversible and specific recognition and adsorption by susceptible bacteria, determining the host specificity and phage infection process [83]. Thus, the differences in the tail of both tested phages in this study, may justify the different obtained results of phage host range. Phage phPA-G is a siphovirus with long, flexible, non-contractile tails. Phage phPA-Intesti is a myovirus that has a long, rigid, contractile tail with a sheath around a central tube. Infection begins through phage recognition and adsorption to a host cell receptor. The adhesion specificity is mediated by the specific recognition between the phage receptor-binding proteins located at the tip of the tail and a receptor located on the surface of the host cell [84]. Thus, tail-related genes have a very important role in host specificity. Considering the differences observed in the genomes of phages phPA-G and phPA-Intesti regarding tail genes, it is expected that there will be differences in host specificity between both phages, as confirmed with the host range and EOP results. In fact, by analyzing the proteins expressed by the known genes (Supplementary Tables S1 and S2), we can observe that phage phPA-Intesti presents two known baseplate spikes as well as three fiber proteins, with all of them aiding in the host recognition process. In contrast, phage phPA-G does not present any known spike or fiber proteins. Therefore, these differences may influence the observed host-range differences.

Different bacterial strains may have different phage-binding receptors and, consequently, different phage susceptibility. Plasmids may have the potential to alter the physiological properties of bacteria with regards to the outer membrane structure, which could affect phage infectivity, by influencing the expression of the bacterial membrane proteins, which act as potential bacterial cell surface receptors recognized by phages [85]. Additionally, the modulation of bacterial cell surface receptors by the phage could also impact the adsorption efficiency of the phage, which leads to varying phage infectivity outcomes [85]. In this study, different *P. aeruginosa* strains harboring different plasmids may result in varying infection efficiency by the two tested phages. Further studies to understand the influence of plasmids towards overall expression of bacteria membrane proteins and, consequently, in the recognition of bacteria by phages would be important. Furthermore, the low EOP values observed for several *P. aeruginosa* strains may also be attributed to the bacterial resistance mechanisms against phage infection. These include several antiviral mechanisms employed by the host cell to evade infection by phages, such as CRISPR [86].

Other *P. aeruginosa* phages also exhibit narrow host specificity, infecting only the original host bacterium or strains of a particular bacterial species [87,88,89,90]. The use of phages with a narrow host range may be advantageous since it would not affect endogenous bacteria other than the target pathogenic bacterium. Moreover, a combination of different narrow-host-range phages can be used to effectively control pathogenic bacteria [91,92]. Another possible strategy to increase the host range of phages is the Appelman protocol [93]. However, regardless of the host range, it is always necessary to test phages against the clinical isolates before application to ensure appropriate and accurate treatment.

Several studies have demonstrated that *P. aeruginosa* inactivation either increases with increasing MOI values or occurs sooner for higher MOI values [88,94,95,96,97]. The MOI can influence the efficiency of the phage and affect the bacterial phage population dynamics by influencing the mutation rate and the overall growth of resistant mutants [98]. Therefore, testing multiple phage concentrations is critical to designing the best treatment. The present study demonstrated that an increase in the MOI from 1 to 100 promoted a significant increase in the efficacy of phage phPA-G. For phage phPA-Intesti, the efficiency was similar for all MOIs. A lower MOI (MOI 1) led to a similar reduction to that of the highest tested MOI (MOI 100). Also, higher phage phPA-Intesti replication was observed at the lowest MOI (MOI 1). These results emphasize the potential of this phage to be applied in phage therapy, since a low phage dose (and therefore less associated costs) is enough to lead to a considerable bacterial reduction. Considering phage replication at this MOI, a single dose of the phage may also be sufficient for effective phage therapy. Considering these results, the biofilm experiments were performed at MOI 1. Similar results were obtained by Vieira et al. (2012) [99] with a specific phage for *P. aeruginosa*. In this study, the reduction in the MOI from 50 to 10 did not significantly reduce phage efficiency in *P. aeruginosa* control. Although bacterial reduction with phage phPA-Intesti occurs earlier at MOI 100 (with a decrease of 1.1 log CFU/mL after 2 h of incubation), the initial doses of phage phPA-Intesti were not essential due to the self-sustaining nature of the phage, as evidenced by the increase in phage titers in the presence of its host at MOI 1. The phage titer during the 12 h of incubation in the presence of the host increased more at MOI 1 (by 3.5 log PFU/mL) than at MOI 100 (by 1.0 log PFU/mL). For phage phPA-G, the number of phage particles during 12 h of incubation in the presence of the *P. aeruginosa* at MOIs of 1, 10, and 100 remained constant (i.e., phage concentration remained similar to that of phage control), showing that the initial doses of this phage were essential for *P. aeruginosa* inactivation.

The phage phPA-Intesti was more efficient than the phage phPA-G; however, none of the phages prevented bacterial regrowth. Some authors have proposed that bacterial regrowth following phage treatment can be prevented by using a cocktail of different lytic phages [57,58,60,100,101,102]. Given the considerable diversity in bacterial surface receptors for phage adsorption, it is imperative to employ a phage cocktail comprising distinct phage particles with varying adsorption mechanisms to maintain phage infectivity [47]. In this study, the phage cocktail (with a maximum reduction of 4.2 log CFU/mL) was more efficient at inactivating *P. aeruginosa* and controlling bacterial regrowth than phage phPA-G alone (with a maximum reduction of 2.6 log CFU/mL). These results are in accordance with other studies [71,103,104], which demonstrated a greater reduction in bacterial populations when phage cocktails were employed than when the single phage suspensions were used. However, phage phPA-Intesti (with a maximum reduction of 4.9 log CFU/mL) was more efficient than phage phPA-G alone and the phage cocktail. When phage phPA-Intesti was used, in general, the bacterial regrowth was lower than with phage phPA-G and the phage cocktail. This could be the result of the phages’ competition for receptors or the establishment of prophages promoting phage resistance [105,106,107]. The genome analysis of phage phPA-G revealed the presence of genes coding for lysogenic markers, such as an integrase and an excisionase, suggesting its potential for establishing a latent infection within host cells under a lysogenic cycle. Manifestations of this life cycle could be observed during the individual phage application, where the bacterial cells started to lose fitness compared to the control group but never reached a point of lysis (no increase in phage titer) [107,108]. Therefore, the prophages could be preventing secondary infections by the lytic phage phPA-Intesti [106] and therefore explain the reduced inactivation of the cocktail. Nonetheless, synergy has been observed when temperate phages have their SOS mechanisms induced by antibiotics [109]. In the future, it would be interesting to evaluate the combination of ciprofloxacin–phPA-G or the combination of ciprofloxacin and phage cocktails in controlling *E. coli*. In the present study, we decided to exclude the temperate phage from further testing and use only the lytic phage, phPA-Intesti.

The analysis of the phage genome is critical to the success of phage therapy. The primary determinants for the therapeutic selection of phages include phage life cycle genes such as integrase genes and the absence of deleterious genes, as well as the presence/absence of phage genes encoding virulence and transducible elements such as antibiotic resistance genes. Our analysis did not detect known genes encoding antibiotic resistance or virulence determinants in the phPA-G and phPA-Intesti genomes. The absence of the phage’s integrase gene and other genes coding for lysogenic markers in the genome of phage phPA-Intesti indicates that this phage could potentially be exploited for biocontrol applications. The presence of an integrase gene is undesirable and nullifies the potential therapeutic use of phage phPA-G. The integrase enzyme could inadvertently integrate phage DNA into the bacterial genome, allowing phage DNA to passively replicate while its host cell continues to divide, delaying host lysis and potentially increasing host resistance to the immune system [110]. Although temperate phages are viable for the delivery of some non-lytic antimicrobial treatments [111,112,113], the risks associated with temperate phages or prophages are sufficient reasons to avoid their use in phage therapies. In the future, this phage could be modified to remove the factors that allow lysogeny, such as integrases and repressors. This approach was employed to treat a patient with a disseminated drug-resistant *Mycobacterium abscessus*, representing the inaugural application of phage therapy utilizing an engineered phage [114]. It will be also important to sequence the genome of the host strain used in this study and evaluate the eventual presence of active prophages, since these integrated prophages can be released to the outside when the host cell lyses after lytic phage infection.

The efficiency of phage phPA-Intesti was tested in human urine samples to evaluate the potential application of this phage for the inactivation of UTIs caused by *P. aeruginosa* biofilms. It was observed that phage phPA-Intesti could successfully inactivate *P. aeruginosa* in urine (maximum inactivation of 5.5 log CFU/cm^2^). The concentration of phage phPA-Intesti remained constant in the absence of the host, indicating that its efficiency in inactivating bacteria is not affected by low pH. The inactivation of *P. aeruginosa* in the urine (maximum reduction of 5.4 log CFU/cm^2^ after 4 h incubation) was similar to that in Tryptic Soy Broth (TSB) (maximum inactivation of 4.9 log CFU/cm^2^ after 8 h incubation). In fact, phage phPA-Intesti replication in the presence of *P. aeruginosa* was similar in the TSB medium and urine (3.5 and 3.2 log PFU/cm^2^, respectively). Pereira et al. (2016) reported that single phage suspensions (E-2 and E-4) and phage cocktail E-2/E-4 reduced approximately 2.0 log CFU/mL of *Enterobacter cloacae* in urine. Duarte et al. (2024) observed that the phage VB_KPM_KP1LMA could successfully inactivate *Klebsiella pneumoniae* in urine with a maximum inactivation of 3.8 log CFU/mL. In these two studies, the efficacy of the phages in the urine was lower than in the liquid medium [71,77], confirming the importance of the characterization of the phages before their application against pathogenic bacteria in adverse environments.

In this study, the combination of phages with ciprofloxacin at MIC neither increased the efficacy of bacterial inactivation compared to treatment with phage phPA-Intesti nor prevented *P. aeruginosa* regrowth. The treatment with phage phPA-Intesti alone presented better results in terms of bacterial inactivation and bacterial regrowth, followed by the treatment using the phage and the antibiotic, as well as treatment with only the antibiotic. The biofilm structure can affect the results of the combination of ciprofloxacin with the phage. The formation of biofilms by *P. aeruginosa* results in the development of multiple antibiotic resistance mechanisms, which represents a significant challenge to the efficacy of conventional single antibiotic therapeutic approaches [115]. The effectiveness of phage–antibiotic combinations also depends on the doses used. In a study conducted by Hong et al. (2024), MOIs of 0.1 and 100 (10^3^, 10^5^, and 10^8^ PFU/mL) were combined with ciprofloxacin (ranging from 1/8× MIC to 8× MIC) for the control of *P. aeruginosa*. These researchers observed that the combination of phage PEV31 and ciprofloxacin demonstrated a heightened bactericidal effect when the ciprofloxacin dosage exceeded 1× MIC at the MOIs of 0.1 and 100 [116]. In the future, it will be important to determine the optimal dosage of phage phPA-Intesti and ciprofloxacin to establish the ideal protocol for an effective treatment against *P. aeruginosa*. These findings will be crucial in guiding the future use of phage–ciprofloxacin formulations for the treatment of UTIs. The time of administration and the number of doses used can also affect the effectiveness of treatment. In this study, the phage and the antibiotic were given at the same time, which may have made them less effective because they both target the same bacterial cells at the same time. The sequential administration of the antibiotic after the phage allows the phage to replicate more effectively, reducing the integrity of the bacterial cells. This can improve antibiotic penetration and prevent bacterial regrowth [117]. The use of a single dose of both antimicrobial agents may explain the rapid regrowth of bacteria observed in the ciprofloxacin and the combined tests. In the future, the sequential approach of adding the antibiotic after the phage and the application of repeated doses of antibiotic must be evaluated.

The effectiveness of the phage phPA-Intesti decreases by the application of ciprofloxacin. Ciprofloxacin binds to either DNA gyrase or topoisomerase IV, preventing the unlinking of DNA following replication. This results in the DNA being unable to unwind correctly, which in turn impairs its normal function during replication and transcription, and can lead to cell death [118]. This affects bacteria replication and, consequently, the replication of phages that require the host’s biochemical machinery for this process. Furthermore, ciprofloxacin not only targets bacterial topoisomerase but also inhibits phage topoisomerase thus preventing viral replication [119,120]. However, in this study, the concentration of virus particles was similar in the presence and absence of antibiotics, which means that there may be other factors influencing the results.

The combination of ciprofloxacin and phage phPA-Intesti did not result in enhanced biofilm cell inactivation, yet the development of resistant bacteria was observed to be less pronounced compared to treatment with the antibiotic alone. Despite the better results using phage phPA-Intesti alone, it has been demonstrated that phage-resistant bacterial strains can become re-sensitized to antibiotics as a result of modifications to the virulence factors on the bacterial cell surface, which are targeted by phages. These modifications are designed to prevent phage adsorption [121]. Thus, strains that may develop resistance to phage treatment may be eliminated with phage and antibiotic treatment. According to Li et al. (2021), the combination of phages and antibiotics represents a promising strategy for reducing the dose of antibiotics and the development of antibiotic resistance during treatment [75]. In fact, in this study, higher bacterial reduction and lower bacterial regrowth were observed with the combination of phage phPA-Intesti and ciprofloxacin compared to the treatment with the antibiotic alone. This, together with the daily use of antibiotics, can facilitate the introduction of phages in the clinic routine when combined with antibiotics. However, it is important to carefully choose the antibiotic and phage to be combined in order to take advantage of the best of each and have an effective treatment. In the future, it would be important to test bacterial-phage-resistant mutants against ciprofloxacin in order to ascertain whether the phage resistance has resulted in a reduction in the antibiotic MIC.

## 5. Conclusions and Future Perspectives

The results highlight the potential of phage phPA-Intesti to inactivate planktonic and biofilm-associated *P. aeruginosa* cells, suggesting that this approach can be used to control UTIs caused by this species. Phage phPA-Intesti is an effective and safe phage that can control *P. aeruginosa* in culture medium and urine and does not suffer inactivation at low pHs.

Although the combined treatment with phage phPA-Intesti and ciprofloxacin at the MIC did not improve the efficacy of bacterial inactivation nor reduce the development of resistant mutants compared with the treatment using only the phage, the development of resistant bacteria was lower in the combined treatment compared to the antibiotic treatment.

In the future, it would be important to understand the interaction between the phages and ciprofloxacin, namely the impact on DNA and phage replication. Also, further studies on the potential of the sequential application of phages and antibiotics could also help improve the results by reducing any antagonistic effect. Moreover, since it has been observed that, in some cases, phages can lead to a reduction in MIC, evaluating the potential of this phage to reduce ciprofloxacin MIC could also lead to new therapeutical potential. A full understanding of these interactions will allow for the formulation of therapeutical protocols and ensure the success of phage therapy in UTIs. Also, the exploitation of the compatibility of phage phPA-Intesti with other phages and antibiotics in order to potentially enable their use against other *P. aeruginosa* strains involved in UTIs needs to be addressed.

## Figures and Tables

**Table 1:** Host range and EOP for phages phPA-G and phPA-Intesti on 14 different *P. aeruginosa* strains. Clear lysis zone (+) and absence of lysis zone (−). The plating with the host strain was considered as EOP = 1. An EOP value of 0 indicates that the phage does not infect the tested bacterial strain (absence of clear lysis zones).

*P. aeruginosa Strains*	Phage phPA-G		Phage phPA-Intesti	
	Spot Test	EOP	Spot Test	EOP
CHUC	+	1 (host)	+	1 (host)
ATCC 27853	−	0	+	0
PI24561	−	0	−	0
IU96131	−	0	−	0
IR82433	−	0	+	3.1 × 10^−2^ ± 1.7 × 10^−2^
IU4506	−	0	−	0
2515567	−	0	−	0
IR83610	−	0	+	4.3 × 10^−1^ ± 1.6 × 10^−1^
IU96174	−	0	−	0
IR80028	−	0	−	0
C563488	−	0	+	0
IR80722	−	0	−	0
IR87252	−	0	−	0
IR77021	−	0	−	0

EOP—efficiency of plating; *P. aeruginosa*—*Pseudomonas aeruginosa*; CHUC—Centro Hospitalar e Universitário de Coimbra; ATCC—American Type Culture Collection.

## Data Availability

Data are contained within the article and Supplementary Materials.

## Supplementary Materials

The following supporting information can be downloaded at: https://www.mdpi.com/article/10.3390/microorganisms12091795/s1, Table S1. Coding sequences identified in phage phPA-G. Table S2. Coding sequences identified in phage phPA-Intesti.
