# Supplementary material for: The Potential of Phage Treatment to Inactivate Planktonic and Biofilm-Forming Pseudomonas aeruginosa
Source: Microorganisms. 2024 Aug 29;12(9):1795. doi: 10.3390/microorganisms12091795 (PMC11433742; doi:10.3390/microorganisms12091795)
Supplement: Supplementary file 1 [file microorganisms-12-01795-s001.zip › microorganisms-3172751-supplementary.pdf]

Table S1. Coding sequences identified in phPA-G phage.

gene	start	stop	frame	Region	Putative function	PHROGs category
WYPXQRSL_0001	1692	1	-	CDS	terminase large subunit	head and packaging
WYPXQRSL_0002	2074	1694	-	CDS	terminase small subunit	head and packaging
WYPXQRSL_0003	2601	2287	-	CDS	HNH endonuclease	DNA, RNA and nucleotide metabolism
WYPXQRSL_0004	3087	2836	-	CDS	hypothetical protein	unknown function
WYPXQRSL_0005	3389	3087	-	CDS	hypothetical protein	unknown function
WYPXQRSL_0006	3507	3379	-	CDS	hypothetical protein	unknown function
WYPXQRSL_0007	3740	3507	-	CDS	hypothetical protein	unknown function
WYPXQRSL_0008	4024	3740	-	CDS	holin	lysis
WYPXQRSL_0009	4421	4017	-	CDS	holin	lysis
WYPXQRSL_0010	5850	5164	-	CDS	hypothetical protein	unknown function
WYPXQRSL_0011	6428	5847	-	CDS	NinG/ Rap DNA junction specific endonuclease	DNA, RNA and nucleotide metabolism
WYPXQRSL_0012	6658	6425	-	CDS	hypothetical protein	unknown function
WYPXQRSL_0013	6924	6655	-	CDS	hypothetical protein	unknown function
WYPXQRSL_0014	7322	6921	-	CDS	NinB/ Orf homologous recombination mediator	DNA, RNA and nucleotide metabolism
WYPXQRSL_0015	7590	7315	-	CDS	hypothetical protein	unknown function
WYPXQRSL_0016	7834	7583	-	CDS	hypothetical protein	unknown function
WYPXQRSL_0017	8031	7834	-	CDS	protease	other
WYPXQRSL_0018	8231	8031	-	CDS	DksA-like zinc-finger protein	other
WYPXQRSL_0019	8715	8233	-	CDS	hypothetical protein	unknown function
WYPXQRSL_0020	8861	8739	-	CDS	hypothetical protein	unknown function
WYPXQRSL_0021	10687	8858	-	CDS	DNA primase/helicase	DNA, RNA and nucleotide metabolism
WYPXQRSL_0022	11601	10684	-	CDS	primosomal protein	DNA, RNA and nucleotide metabolism
WYPXQRSL_0023	11786	11598	-	CDS	hypothetical protein	unknown function

WYPXQRSL_0024	11980	11768	-	CDS	hypothetical protein	unknown function
WYPXQRSL_0025	12555	11983	-	CDS	CII-like transcriptional activator	transcription regulation
WYPXQRSL_0026	12791	12591	-	CDS	hypothetical protein	unknown function
WYPXQRSL_0027	12828	13679	+	CDS	CI-like repressor	transcription regulation
WYPXQRSL_0028	13709	14359	+	CDS	hypothetical protein	unknown function
WYPXQRSL_0029	15030	15785	+	CDS	hypothetical protein	unknown function
WYPXQRSL_0030	16599	16805	+	CDS	hypothetical protein	unknown function
WYPXQRSL_0031	16816	17022	+	CDS	hypothetical protein	unknown function
WYPXQRSL_0032	17019	17228	+	CDS	hypothetical protein	unknown function
WYPXQRSL_0033	17308	17490	+	CDS	hypothetical protein	unknown function
WYPXQRSL_0034	17736	18227	+	CDS	hypothetical protein	unknown function
WYPXQRSL_0035	18345	18929	+	CDS	hypothetical protein	unknown function
WYPXQRSL_0036	18968	19324	+	CDS	Avd protein of DGR	DNA, RNA and nucleotide metabolism
WYPXQRSL_0037	19597	20706	+	CDS	Reverse transcriptase	DNA, RNA and nucleotide metabolism
WYPXQRSL_0038	20811	21107	+	CDS	hypothetical protein	unknown function
WYPXQRSL_0039	21142	21555	+	CDS	hypothetical protein	unknown function
WYPXQRSL_0040	21863	22237	+	CDS	hypothetical protein	unknown function
WYPXQRSL_0041	22234	22767	+	CDS	hypothetical protein	unknown function
WYPXQRSL_0042	22764	23051	+	CDS	hypothetical protein	unknown function
WYPXQRSL_0043	23048	23161	+	CDS	hypothetical protein	unknown function
WYPXQRSL_0044	23175	23903	+	CDS	hypothetical protein	unknown function
WYPXQRSL_0045	23900	24037	+	CDS	hypothetical protein	unknown function
WYPXQRSL_0046	24188	24640	+	CDS	hypothetical protein	unknown function
WYPXQRSL_0047	24685	25218	+	CDS	hypothetical protein	unknown function
WYPXQRSL_0048	25187	25804	+	CDS	exonuclease	DNA, RNA and nucleotide metabolism
WYPXQRSL_0049	25808	26089	+	CDS	hypothetical protein	unknown function
WYPXQRSL_0050	26153	26752	+	CDS	hypothetical protein	unknown function
WYPXQRSL_0051	26915	27139	+	CDS	hypothetical protein	unknown function

WYPXQRSL_0052	27136	28248	+	CDS	hypothetical protein	unknown function
WYPXQRSL_0053	28248	28748	+	CDS	DNA methyltransferase	other
WYPXQRSL_0054	28745	29287	+	CDS	Lar-like restriction alleviation protein	moron, auxiliary metabolic gene and host takeover
WYPXQRSL_0055	29371	29517	+	CDS	hypothetical protein	unknown function
WYPXQRSL_0056	29502	29987	+	CDS	hypothetical protein	unknown function
WYPXQRSL_0057	29990	30304	+	CDS	hypothetical protein	unknown function
WYPXQRSL_0058	30301	30519	+	CDS	hypothetical protein	unknown function
WYPXQRSL_0059	30516	30794	+	CDS	hypothetical protein	unknown function
WYPXQRSL_0060	30791	31180	+	CDS	hypothetical protein	unknown function
WYPXQRSL_0061	31143	31301	+	CDS	hypothetical protein	unknown function
WYPXQRSL_0062	31298	31642	+	CDS	hypothetical protein	unknown function
WYPXQRSL_0063	31818	32132	+	CDS	excisionase	integration and excision
WYPXQRSL_0064	32171	33127	+	CDS	integrase	integration and excision
WYPXQRSL_0065	33586	33323	-	CDS	hypothetical protein	unknown function
WYPXQRSL_0066	33951	33622	-	CDS	hypothetical protein	unknown function
WYPXQRSL_0067	34316	33948	-	CDS	hypothetical protein	unknown function
WYPXQRSL_0068	34942	34313	-	CDS	endolysin	lysis
WYPXQRSL_0069	35285	35172	-	CDS	hypothetical protein	unknown function
WYPXQRSL_0070	36655	35381	-	CDS	hypothetical protein	unknown function
WYPXQRSL_0071	38969	36717	-	CDS	hypothetical protein	unknown function
WYPXQRSL_0072	41758	39029	-	CDS	hypothetical protein	unknown function
WYPXQRSL_0073	42137	41730	-	CDS	minor tail protein	tail
WYPXQRSL_0074	42272	42511	+	CDS	hypothetical protein	unknown function
WYPXQRSL_0075	43178	42687	-	CDS	minor tail protein	tail
WYPXQRSL_0076	43641	43162	-	CDS	virion structural protein	head and packaging
WYPXQRSL_0077	46007	43638	-	CDS	tail length tape measure protein	tail
WYPXQRSL_0078	46310	46056	-	CDS	tail protein	tail
WYPXQRSL_0079	46693	46334	-	CDS	tail assembly chaperone	tail

WYPXQRSL_0080	47224	46703	-	CDS	major tail protein	tail
WYPXQRSL_0081	47664	47299	-	CDS	tail terminator	connector
WYPXQRSL_0082	48161	47667	-	CDS	hypothetical protein	unknown function
WYPXQRSL_0083	48771	48154	-	CDS	methyltransferase	other
WYPXQRSL_0084	48953	48768	-	CDS	hypothetical protein	unknown function
WYPXQRSL_0085	49783	48950	-	CDS	hypothetical protein	unknown function
WYPXQRSL_0086	50148	49792	-	CDS	head closure Hc1	connector
WYPXQRSL_0087	50469	50149	-	CDS	head-tail adaptor Ad1	connector
WYPXQRSL_0088	50854	50450	-	CDS	alpha-2;3-sialyltransferase	other
WYPXQRSL_0089	52400	51213	-	CDS	major head protein	head and packaging
WYPXQRSL_0090	53287	52397	-	CDS	head maturation protease	head and packaging
WYPXQRSL_0091	54681	53419	-	CDS	portal protein	head and packaging
WYPXQRSL_0092	54832	54674	-	CDS	hypothetical protein	unknown function

Table S2. Coding sequences in phPA-Intesti phage.

gene	start	stop	frame	Region	Putative function	PHROGs category
TQVEPAJM_0001	1	1419	+	CDS	terminase large subunit	head and packaging
TQVEPAJM_0002	1430	2917	+	CDS	portal protein	head and packaging
TQVEPAJM_0003	2919	3335	+	CDS	DNA methyltransferase	other
TQVEPAJM_0004	3328	4215	+	CDS	hypothetical protein	unknown function
TQVEPAJM_0005	4232	4606	+	CDS	hypothetical protein	unknown function
TQVEPAJM_0006	4617	5672	+	CDS	major head protein	head and packaging
TQVEPAJM_0007	5726	6211	+	CDS	hypothetical protein	unknown function
TQVEPAJM_0008	6189	6656	+	CDS	hypothetical protein	unknown function
TQVEPAJM_0009	6656	7027	+	CDS	head protein	head and packaging
TQVEPAJM_0010	7027	7647	+	CDS	hypothetical protein	unknown function
TQVEPAJM_0011	7650	8972	+	CDS	tail sheath	tail
TQVEPAJM_0012	8983	9504	+	CDS	virion structural protein	head and packaging
TQVEPAJM_0013	9557	10060	+	CDS	virion structural protein	head and packaging
TQVEPAJM_0014	10060	10563	+	CDS	virion structural protein	head and packaging
TQVEPAJM_0015	10577	10963	+	CDS	tail assembly chaperone	tail
TQVEPAJM_0016	10984	11205	+	CDS	hypothetical protein	unknown function
TQVEPAJM_0017	11195	13531	+	CDS	tail length tape measure protein	tail
TQVEPAJM_0018	13528	14295	+	CDS	tail fiber protein	tail
TQVEPAJM_0019	14295	14660	+	CDS	virion structural protein	head and packaging
TQVEPAJM_0020	14661	15542	+	CDS	baseplate hub	tail
TQVEPAJM_0021	15539	16297	+	CDS	baseplate spike	tail
TQVEPAJM_0022	16304	16669	+	CDS	baseplate wedge subunit	tail
TQVEPAJM_0023	16670	18136	+	CDS	baseplate wedge subunit	tail
TQVEPAJM_0024	18138	18902	+	CDS	structural protein	head and packaging
TQVEPAJM_0025	18912	20933	+	CDS	tail fiber protein	tail

TQVEPAJM_0026	20979	21356	+	CDS	tail fiber assembly	tail
TQVEPAJM_0027	21366	22859	+	CDS	tail fiber protein	tail
TQVEPAJM_0028	22861	23424	+	CDS	endolysin	head and packaging
TQVEPAJM_0029	23405	23671	+	CDS	hypothetical protein	unknown function
TQVEPAJM_0030	23668	24084	+	CDS	Rz-like spanin	lysis
TQVEPAJM_0031	24234	24533	+	CDS	hypothetical protein	unknown function
TQVEPAJM_0032	24549	24836	+	CDS	hypothetical protein	unknown function
TQVEPAJM_0033	24833	24952	+	CDS	hypothetical protein	unknown function
TQVEPAJM_0034	24945	25364	+	CDS	hypothetical protein	unknown function
TQVEPAJM_0035	25388	25597	+	CDS	hypothetical protein	unknown function
TQVEPAJM_0036	25594	25971	+	CDS	hypothetical protein	unknown function
TQVEPAJM_0037	26201	26401	+	CDS	hypothetical protein	unknown function
TQVEPAJM_0038	26412	26678	+	CDS	hypothetical protein	unknown function
TQVEPAJM_0039	26675	27043	+	CDS	hypothetical protein	unknown function
TQVEPAJM_0040	27053	27904	+	CDS	lipoprotein	other
TQVEPAJM_0041	27953	28273	+	CDS	hypothetical protein	unknown function
TQVEPAJM_0042	28275	28646	+	CDS	hypothetical protein	unknown function
TQVEPAJM_0043	28639	29859	+	CDS	RNA ligase	DNA, RNA and nucleotide metabolism
TQVEPAJM_0044	29869	30054	+	CDS	hypothetical protein	unknown function
TQVEPAJM_0045	30095	30490	+	CDS	hypothetical protein	unknown function
TQVEPAJM_0046	30477	31013	+	CDS	hypothetical protein	unknown function
TQVEPAJM_0047	31013	31279	+	CDS	hypothetical protein	unknown function
TQVEPAJM_0048	31329	33281	+	CDS	DNA primase/helicase	DNA, RNA and nucleotide metabolism
TQVEPAJM_0049	33302	35959	+	CDS	DNA polymerase I	DNA, RNA and nucleotide metabolism
TQVEPAJM_0050	35976	36182	+	CDS	hypothetical protein	unknown function
TQVEPAJM_0051	36329	36514	+	CDS	hypothetical protein	unknown function
TQVEPAJM_0052	36582	37475	+	CDS	hypothetical protein	unknown function
TQVEPAJM_0053	37534	37728	+	CDS	hypothetical protein	unknown function

TQVEPAJM_0054	37729	38004	+	CDS	hypothetical protein	unknown function
TQVEPAJM_0055	38016	38282	+	CDS	hypothetical protein	unknown function
TQVEPAJM_0056	38282	39370	+	CDS	exonuclease	DNA, RNA and nucleotide metabolism
TQVEPAJM_0057	39358	39519	+	CDS	hypothetical protein	unknown function
TQVEPAJM_0058	39519	39692	+	CDS	hypothetical protein	unknown function
TQVEPAJM_0059	39689	40252	+	CDS	HNH endonuclease	DNA, RNA and nucleotide metabolism
TQVEPAJM_0060	40249	40464	+	CDS	hypothetical protein	unknown function
TQVEPAJM_0061	40474	41391	+	CDS	DNA polymerase exonuclease subunit	DNA, RNA and nucleotide metabolism
TQVEPAJM_0062	41445	41669	+	CDS	hypothetical protein	unknown function
TQVEPAJM_0063	41666	42079	+	CDS	hypothetical protein	unknown function
TQVEPAJM_0064	42083	42982	+	CDS	thymidylate synthase	DNA, RNA and nucleotide metabolism
TQVEPAJM_0065	42986	43360	+	CDS	thymidylate synthase	DNA, RNA and nucleotide metabolism
TQVEPAJM_0066	43353	45209	+	CDS	ribonucleoside-diphosphate reductase large subunit	DNA, RNA and nucleotide metabolism
TQVEPAJM_0067	45219	45584	+	CDS	hypothetical protein	unknown function
TQVEPAJM_0068	45626	46645	+	CDS	ribonucleoside diphosphate reductase small subunit	DNA, RNA and nucleotide metabolism
TQVEPAJM_0069	46706	47188	+	CDS	unknown function	unknown function
TQVEPAJM_0070	47178	47441	+	CDS	hypothetical protein	unknown function
TQVEPAJM_0071	47434	47664	+	CDS	hypothetical protein	unknown function
TQVEPAJM_0072	47666	47908	+	CDS	hypothetical protein	unknown function
TQVEPAJM_0073	47960	48514	+	CDS	hypothetical protein	unknown function
TQVEPAJM_0074	48495	48668	+	CDS	hypothetical protein	unknown function
TQVEPAJM_0075	48678	49007	+	CDS	hypothetical protein	unknown function
TQVEPAJM_0076	49007	49174	+	CDS	hypothetical protein	unknown function
TQVEPAJM_0077	49227	49724	+	CDS	hypothetical protein	unknown function
TQVEPAJM_0078	49721	50200	+	CDS	hypothetical protein	unknown function
TQVEPAJM_0079	51096	51365	+	CDS	hypothetical protein	unknown function
TQVEPAJM_0080	51377	51610	+	CDS	hypothetical protein	unknown function
TQVEPAJM_0081	51669	51872	+	CDS	hypothetical protein	unknown function

TQVEPAJM_0082	51948	52496	+	CDS	hypothetical protein	unknown function
TQVEPAJM_0083	52577	52975	+	CDS	hypothetical protein	unknown function
TQVEPAJM_0084	53042	53431	+	CDS	hypothetical protein	unknown function
TQVEPAJM_0085	53456	53569	+	CDS	hypothetical protein	unknown function
TQVEPAJM_0086	53670	53804	+	CDS	hypothetical protein	unknown function
TQVEPAJM_0087	53789	54181	+	CDS	hypothetical protein	unknown function
TQVEPAJM_0088	54213	54533	+	CDS	hypothetical protein	unknown function
TQVEPAJM_0089	54533	54769	+	CDS	hypothetical protein	unknown function
TQVEPAJM_0090	54771	55049	+	CDS	hypothetical protein	unknown function
TQVEPAJM_0091	55173	55409	+	CDS	hypothetical protein	unknown function
TQVEPAJM_0092	55557	56048	+	CDS	hypothetical protein	unknown function
TQVEPAJM_0093	56059	56418	+	CDS	hypothetical protein	unknown function
TQVEPAJM_0094	56444	56911	+	CDS	hypothetical protein	unknown function
TQVEPAJM_0095	56995	57240	+	CDS	hypothetical protein	unknown function
TQVEPAJM_0096	57316	57849	+	CDS	hypothetical protein	unknown function
TQVEPAJM_0097	57935	58450	+	CDS	hypothetical protein	unknown function
TQVEPAJM_0098	58688	58900	+	CDS	hypothetical protein	unknown function
TQVEPAJM_0099	58926	59315	+	CDS	hypothetical protein	unknown function
TQVEPAJM_0100	59388	59903	+	CDS	hypothetical protein	unknown function
TQVEPAJM_0101	59973	60167	+	CDS	hypothetical protein	unknown function
TQVEPAJM_0102	60403	60690	+	CDS	hypothetical protein	unknown function
TQVEPAJM_0103	60772	61026	+	CDS	hypothetical protein	unknown function
TQVEPAJM_0104	61440	61658	+	CDS	hypothetical protein	unknown function
TQVEPAJM_0105	61648	61872	+	CDS	hypothetical protein	unknown function
TQVEPAJM_0106	63211	63642	+	CDS	hypothetical protein	unknown function
TQVEPAJM_0107	63635	63973	+	CDS	hypothetical protein	unknown function
TQVEPAJM_0108	63963	64337	+	CDS	hypothetical protein	unknown function
TQVEPAJM_0109	64324	64686	+	CDS	hypothetical protein	unknown function

TQVEPAJM_0110	64673	65014	+	CDS	hypothetical protein	unknown function
TQVEPAJM_0111	65011	65913	+	CDS	hypothetical protein	unknown function
TQVEPAJM_0112	65910	66263	+	CDS	hypothetical protein	unknown function
TQVEPAJM_0113	66263	66943	+	CDS	hypothetical protein	unknown function
TQVEPAJM_0114	66953	67300	+	CDS	hypothetical protein	unknown function
TQVEPAJM_0115	67293	67517	+	CDS	hypothetical protein	unknown function
TQVEPAJM_0116	67504	67818	+	CDS	hypothetical protein	unknown function
TQVEPAJM_0117	67856	68044	+	CDS	hypothetical protein	unknown function
TQVEPAJM_0118	68141	68530	+	CDS	hypothetical protein	unknown function
TQVEPAJM_0119	68530	69246	+	CDS	hypothetical protein	unknown function
TQVEPAJM_0120	69239	69484	+	CDS	hypothetical protein	unknown function
TQVEPAJM_0121	69484	69729	+	CDS	hypothetical protein	unknown function
TQVEPAJM_0122	69773	70285	+	CDS	hypothetical protein	unknown function
TQVEPAJM_0123	70278	70607	+	CDS	hypothetical protein	unknown function
TQVEPAJM_0124	70600	70794	+	CDS	hypothetical protein	unknown function
TQVEPAJM_0125	70791	71267	+	CDS	hypothetical protein	unknown function
TQVEPAJM_0126	71248	71748	+	CDS	hypothetical protein	unknown function
TQVEPAJM_0127	71832	72089	+	CDS	hypothetical protein	unknown function
TQVEPAJM_0128	72073	72222	+	CDS	hypothetical protein	unknown function
TQVEPAJM_0129	72219	72587	+	CDS	hypothetical protein	unknown function
TQVEPAJM_0130	73364	73140	-	CDS	hypothetical protein	unknown function
TQVEPAJM_0131	73717	73439	-	CDS	hypothetical protein	unknown function
TQVEPAJM_0132	73893	73714	-	CDS	hypothetical protein	unknown function
TQVEPAJM_0133	74465	73890	-	CDS	hypothetical protein	unknown function
TQVEPAJM_0134	74694	74467	-	CDS	hypothetical protein	unknown function
TQVEPAJM_0135	75026	74691	-	CDS	hypothetical protein	unknown function
TQVEPAJM_0136	75445	75017	-	CDS	peptidase HslV family	other
TQVEPAJM_0137	75648	75442	-	CDS	hypothetical protein	unknown function

TQVEPAJM_0138	75845	75657	-	CDS	hypothetical protein	unknown function
TQVEPAJM_0139	76113	75835	-	CDS	hypothetical protein	unknown function
TQVEPAJM_0140	77672	76110	-	CDS	DNA helicase	DNA, RNA and nucleotide metabolism
TQVEPAJM_0141	78135	77650	-	CDS	hypothetical protein	unknown function
TQVEPAJM_0142	78692	78090	-	CDS	DprA-like DNA recombination-mediator protein	DNA, RNA and nucleotide metabolism
TQVEPAJM_0143	78937	78689	-	CDS	hypothetical protein	unknown function
TQVEPAJM_0144	79510	78947	-	CDS	endolysin	lysis
TQVEPAJM_0145	80057	79563	-	CDS	hypothetical protein	unknown function
TQVEPAJM_0146	81388	80117	-	CDS	ATP-dependent DNA ligase	DNA, RNA and nucleotide metabolism
TQVEPAJM_0147	81673	81401	-	CDS	hypothetical protein	unknown function
TQVEPAJM_0148	82065	81676	-	CDS	dCMP deaminase	DNA, RNA and nucleotide metabolism
TQVEPAJM_0149	82526	82089	-	CDS	hypothetical protein	unknown function
TQVEPAJM_0150	82801	82535	-	CDS	hypothetical protein	unknown function
TQVEPAJM_0151	83309	82911	-	CDS	hypothetical protein	unknown function
TQVEPAJM_0152	83872	83306	-	CDS	hypothetical protein	unknown function
TQVEPAJM_0153	84352	84708	+	CDS	hypothetical protein	unknown function
TQVEPAJM_0154	84710	84958	+	CDS	hypothetical protein	unknown function
TQVEPAJM_0155	85163	85465	+	CDS	hypothetical protein	unknown function
TQVEPAJM_0156	86348	87166	+	CDS	terminase large subunit	head and packaging
TQVEPAJM_0157	87239	87664	+	CDS	head fiber protein	head and packaging
